# Investigating Adherence to the 2022 NICE Vitamin D Management Guidelines on MHSOP (Mental Health Services for Older People) Wards

**DOI:** 10.1192/bjo.2025.10626

**Published:** 2025-06-20

**Authors:** Sooyoung Lee

**Affiliations:** Nottinghamshire Healthcare NHS Foundation Trust, Nottingham, United Kingdom

## Abstract

**Aims:** Vitamin D deficiency is a widespread problem in older people with reduced ability for production, along with risk factors such as indoor living. Improving adherence to the vitamin D guidelines is a simple and cost-effective method of potentially reducing falls, morbidity and mortality in older patients with risk factors.

Aim is to assess whether vitamin D deficiency is identified and managed appropriately according to the 2022 NICE vitamin D guidelines.

Standard:

1. All patients to have vitamin D level tested on admission.

2. All patients with vitamin D level of 50 and below to be prescribed vitamin D as per guidelines.

3. If calcium deficiency co-exists with vitamin D deficiency, patients to be prescribed replacements for both as per guidelines.

4. All patients to be referred for specialist services if vitamin D deficiency presents with the following: eGFR <30, hypercalcaemia, or granulomatous conditions.

**Methods:** The audit was registered with the Trust following discussion with the ward managers and consultants. All inpatients on two MHSOP wards on the day of data collection were included. Using an audit questionnaire, retrospective data was collected from electronic patient notes, paper drug charts and electronic blood reporting system. Data was analysed on Excel. The re-audit occurred six months later following intervention.

Interventions following the initial audit involved designing a vitamin D awareness poster for the wards, meetings with the ward pharmacist and presentation at the local MHSOP clinical effectiveness meeting to raise awareness.

**Results:** First cycle: 34 patients were included. 30 out of 34 (88%) patients had their vitamin D levels tested on admission or had a recent level. Out of the 30 patients who had recent vitamin D levels on record, 15 patients had adequate vitamin D levels, seven had insufficient levels, and eight had deficient levels. Out of the 15 patients who had insufficient or deficient levels, nine patients (60%) were prescribed vitamin D. One patient who required specialist services did not get referred.

Second cycle: 33 patients were included. 31 patients out of 33 (94%) had vitamin D testing on admission. Out of the 31 patients, 12 patients had deficient or insufficient vitamin D levels requiring prescription. Nine out of these 12 patients (75%) were prescribed vitamin D.

**Conclusion:** Following simple interventions to raise awareness, the re-audit results showed improvements in vitamin D testing on admission as well as improved management. Ongoing communication with pharmacists and rotating resident doctors is required to sustain awareness and improve adherence.

